# Pollution, Health, and the Moderating Role of Physical Activity Opportunities

**DOI:** 10.3390/ijerph17176272

**Published:** 2020-08-28

**Authors:** George B. Cunningham, Pamela Wicker, Brian P. McCullough

**Affiliations:** 1 Center for Sport Management Research and Education, Department of Health and Kinesiology, Texas A&M University, College Station, TX 77843-4243, USA; brian.mccullough@tamu.edu; 2 Department of Sports Science, Bielefeld University, 33615 Bielefeld, Germany; pamela.wicker@uni-bielefeld.de

**Keywords:** Air pollution, water pollution, access to physical activity, mental health, physical health

## Abstract

Air and water pollution have detrimental effects on health, while physical activity opportunities have a positive relationship. The purpose of this study was to explore whether physical activity opportunities moderate the relationships among air and water pollution, and measures of health. Aggregate data were collected at the county level in the United States (*n* = 3104). Variables included the mean daily density of fine particle matter (air pollution), reported cases of health-related drinking water violations (water pollution), subjective ratings of poor or fair health (overall health), the number of physically and mentally unhealthy (physical and mental health, respectively), and the percentage of people living in close proximity to a park or recreation facility (access to physical activity). Air and water pollution have a significant positive effect on all measures of residents’ poor health, while physical activity opportunities only have a negative effect on overall health and physical health. Access to physical activity only moderates the relationship between air pollution and all health outcomes. Since physical activity behavior can be more rapidly changed than some causes of pollution, providing the resident population with better access to physical activity can represent an effective tool in environmental health policy.

## 1. Introduction

The Intergovernmental Panel on Climate Change (IPCC) has noted that climate change increases the risks to human security, livelihoods, food security, water supply, and health [1]. These risks are mostly associated with air and water pollution. Human activity and dependence on fossil fuels have led to the exacerbation of carbon in the atmosphere [2]. Similarly, the increases of industrialization, civilization, and living standards have increased the presence of physical, chemical, and biological pollutants, resulting in harmful effects of water pollution [3]. Consistent with the IPCC’s [1] warnings, air and water pollution negatively affect individuals’ health [4,5,6]. Following the Lancet Commission on pollution and health, “pollution is the largest environmental cause of disease and premature death in the world today” [7]

These detrimental impacts of air and water pollutants extend to sport and physical activity, and greater attention is necessary to examine the impacts pollution may have on individuals’ physical activity levels and their health [8]. By way of example, physical inactivity increases as a result of higher levels of air pollution [8], and air pollution and physical inactivity are significant contributors to heart disease and increased mortality [9]. Roberts et al. [8] noted, however, that the causal inference between pollution and physical (in)activity required more investigation.

Such an investigation is also necessary from a policy perspective. For example, the World Health Organization (WHO) recommends regular physical activity for individuals of all age groups because its positive impacts on several relevant health outcomes, including cardiorespiratory and muscular fitness, bone and functional health, reduced risk of non-communicable diseases, and various indices of mental health and well-being [10]. Likewise, the U.S. Department of Health and Human Services [11] has stressed the physical and psychological health benefits of physical activity and encourages the American population to be regularly physically active. This Department also acknowledges that physical activity patterns of the population are not only a result of individual efforts, but that communities “can provide many opportunities for physical activity, such as walking trails, bicycle lanes on roads, sidewalks, and sports fields” [11]. The link between such opportunities for physical activity and actual physical activity has been well documented in the literature [12]. Hence, people who have more possibilities to be active are likely to be active themselves, resulting in improved health. The question is if the positive health effects of physical activity opportunities are still in effect when the area where people live is characterized by high levels of pollution.

The purpose of this study was to explore the influences of air and water pollution and access to physical activity on resident health and the moderating role of physical activity opportunities. This study advances the following two research questions: (a) how do water and air pollution, and physical activity opportunities affect resident health; and (b) does access to physical activity moderate the relationship between water/air pollution and resident health? These research questions will be answered using aggregate data at the county level in the U.S. This approach differs from most previous studies, where the focus was on the effects of pollution on health at the individual level. The study adds to the scant body of knowledge examining the role of physical activity (opportunities) at the intersection of pollution and resident health. The findings have implications for environmental health policy and the promotion of physical activity.

### 1.1. Theoretical Background and Literature Review

#### 1.1.1. Overall Health, Physical Health, and Mental Health

According to the World Health Organization [10], an individual’s overall health status consists of two dimensions: physical health and mental health. Whereas the former is conceptualized as the absence of diseases (e.g., cancer, hypertension, osteoporosis, cardiovascular heart diseases, or type 2 diabetes), the latter is “a state of well-being in which every individual realizes his or her own potential, can cope with the normal stresses of life, can work productively and fruitfully, and is able to make a contribution to his or her community” [10]. Epidemiological studies typically use clinical indicators to capture these two dimensions of health. Research in the social sciences has also used self-reported measures to capture individuals’ overall health [13,14]. Likewise, subjective measures have been applied to capture physical health [15,16] and mental health [15] and its components, such as life satisfaction [17] and happiness [18].

#### 1.1.2. Pollution and Health

The health effects of air and water pollution have been widely studied, with studies consistently documenting a negative impact of pollution on individuals’ physical and mental health [6,7]. For instance, exposure to air pollutants increases the prevalence of respiratory and cardiovascular diseases which, in turn, increase mortality rates and hospital admissions [4,19]. Further health problems resulting from exposure to polluted air include lung cancer, heart and lung diseases, and asthmatic attacks [20]. Furthermore, air pollution can increase premature mortality and reduce life expectancy [20].

Concerning physical health and drinking water pollution, contaminated water increases the prevalence of a number of diseases, such as hepatitis, typhoid fever, gastroenteritis, dysentery, cholera, diarrhea, malaria, giardiasis, and intestinal worms [21]. Further diseases and health problems resulting from water pollution include skin and respiratory illnesses, anemia, yellow fever, and dengue [22]. Complications in childbirth were also reported [22]. Increases in water pollution also result in higher levels of toxicity poisoning and cancer rates in general [5], as well as digestive cancer [23]. Accordingly, residents who were more affected by water pollution reported significantly poorer physical health [15].

Turning to mental health, air pollution can negatively affect several components of individuals’ mental health, such as subjective well-being [24], life satisfaction [25], happiness [6], depressive symptoms, and overall mental health status [26]. Likewise, water pollution is associated with worse mental health outcomes ([15,27]). These well-established relationships between pollution and poor health are covered in our first hypothesis:

**Hypothesis 1a** **(H1a).** *Air pollution will be positively related to residents’ poor health.*

**Hypothesis 1b** **(H1b).** *Water pollution will be positively related to residents’ poor health.*

#### 1.1.3. Physical Activity Opportunities and Health

Previous research has shown that a number of other factors affect individuals’ physical health [28] and mental health [29]. One of these factors is physical activity, which contributes positively to individuals’ physical health [13,30,31] and mental health [32,33]. One important precondition of people’s participation in physical activity are opportunities for sport and physical activity, including parks and sport facilities [34,35] as well as a walkable neighborhood and recreational facilities [36], supporting the overall importance of community design.

Since physical activity opportunities facilitate actual participation in physical activity which, in turn, adds to physical and mental health, physical activity opportunities should also be associated with beneficial health outcomes. However, this association has received less research attention. The few existing studies have supported the notion that access to physical activity opportunities is positively associated with mental health aspects [17,37]. Likewise, physical activity opportunities were found to be positively associated with physical health aspects such as fitness level and weight status [38]. Consequently, the second hypothesis proposes the following relationship:

**Hypothesis 2** **(H2).** *Physical activity opportunities will be negatively related to residents’ poor health.*

#### 1.1.4. The Moderating Role of Physical Activity Opportunities

Existing research has also investigated the link between pollution, physical activity, and health [39,40]. Physical activity has already been shown to have a moderating effect on mental health outcomes in other adverse contexts beyond pollution [16]. In the context of air pollution, Chinese scholars have suggested that outdoor physical activity in polluted conditions can increase the likelihood of health problems, especially in conditions of extreme air pollution [39,41]; however, the researchers have not offered empirical evidence of the relationships. The majority of empirical studies point to beneficial effects of physical activity on health which outweigh the harm caused by air pollution [40,42], supporting the notion of a positive moderating effect. Although the role of physical activity opportunities has not yet been studied, the previous argument stressing the link between opportunities of and actual participation in physical activity [36] is expected to hold here as well. Consequently, we hypothesize that the negative impacts of pollution will be attenuated when more physical activity opportunities are available to residents:

**Hypothesis 3a** **(H3a).** *Physical activity opportunities will moderate the relationship between air pollution and health, such that the effects attenuate as opportunities for physical activity increase.*

**Hypothesis 3b** **(H3b).** *Physical activity opportunities will moderate the relationship between water pollution and health, such that the effects attenuate as opportunities for physical activity increase.*

## 2. Materials and Methods

### 2.1. Data Collection, Measures, and Variables

We collected data at the county level in the U.S. (*N* = 3104). For context, according to the U.S. Census Bureau, in 2019 there are 3141 counties or county-equivalents in the U.S., with an average population of 104,468. All data were publicly available from a variety of sources. Unless otherwise indicated, the County Health Rankings and Roadmaps website (www.countyhealthrankings.org), supported by the Robert Wood Johnson Foundation, provided links for original sources. We drew from their 2020 rankings. Table 1 provides an overview of the variables collected, as well as their descriptive statistics.

#### 2.1.1. Pollution

We used two measures of pollution: air pollution (AirPollution) and drinking water pollution (WaterPollution). We used the mean daily density of fine particle matter in micrograms per cubic meter (PM2.5) to represent AirPollution. These data are available from the Centers for Disease Control and Prevention’s Environmental Public Health Tracking Network (https://www.cdc.gov/nceh/tracking/). Others [43,44] have also used this measure in their analyses. Examination of the data using Box Plots through SPSS (IBM Corp., Armonk, NY., U.S.) identified one extreme outlier: Kern County, CA, with a value of 19.7 (10 greater than the mean). For WaterPollution, we considered whether the county had reported cases of health-related drinking water violations, with data available from the Environmental Protection Agency’s Safe Drinking Water Drinking Information System (https://www3.epa.gov/enviro/facts/sdwis/search.html). Other researchers have also used this source to consider the influence of pollution on health [45,46]. As the measure is dichotomous, we did not check for outliers.

#### 2.1.2. Health

We used three measures of poor health, all from the Behavioral Risk Factor Surveillance System (BRFSS): subjective ratings of poor or fair health (PoorFairHealth), the average number of physically unhealthy days (PhysUnhealthyDays), and the average number of mentally unhealthy days (MenUnhealthyDays) during the last 30 days. These measures capture perceptions of individuals’ overall health status as well as its physical and mental dimensions. The PoorFairHealth ratings comes from a single item scale where participants rate their current overall health from 1 (poor) to 5 (excellent). Others have already used the measure to capture individuals’ subjective health [34,47]. For PhysUnhealthyDays and MenUnhealthyDays, the data are based on responses to separate questions measuring the number of days during the past 30 days that respondents experienced physical (or mental) health that was not good. Others have used these variables in their analysis of physical and mental health [48,49]. For all health-related measures, the County Health Rankings and Roadmaps creates an age-adjusted measure and then uses multi-level regression and post-stratification to generate aggregate estimates for each county (for an overview, see [50,51]).

#### 2.1.3. Physical Activity Opportunities

An original measure from the County Health Rankings and Roadmaps is access to physical activity (AccesstoPA) which represents the percentage of people within a given county who lived in close proximity to a location for physical activity. People are considered to be in close proximity if any of the three conditions apply: (a) they live within a half-mile of a park, (b) they live in an urban area and are within a mile of a recreation facility, or (c) they live in a rural area and are within 3 miles of a recreation facility. Geocodes and census data are used to compute the variable. Parks and recreation facilities represent areas where people can exercise, swim, picnic, ski, golf, participate in formal sport, and the like. According to the U.S. Census Bureau, urban areas are those with 50,000 people or more, whereas rural areas have fewer than 2500 people.

#### 2.1.4. Controls

We included a number of controls that could also influence health. All data were available on the County Health Rankings and Roadmaps website. First, given the negative health effects of smoking [52], we controlled for the percent of county residents who smoked. The Smokers data were gathered from the BRFSS. Weight can also influence health, as people classified as overweight or obese frequently experience poorer health outcomes than do their peers (e.g., [53]). Thus, we controlled for the percent of adults classified as obese (Obesity) by drawing from the United States Diabetes Surveillance System. Food insecurity, which occurs when people lack adequate access to food, can also negatively affect health and well-being [54]. The percent of the county population that experienced food insecurity (FoodInsecure) was captured drawing from the Map the Meal Gap data. A lack of health insurance negatively impacts preventative health and treatment disease [55]; therefore, we controlled for the percent of the county residents who were uninsured (Uninsured) using data from the Small Area Health Insurance Estimates.

Measures of social class, such as educational attainment and household income, can also influence health outcomes, with higher values on each frequently linked with better health [56]. Thus, we controlled for the percent of the county residents who had at least some college education (SomeCollege) using data from the American Community Survey, their household income (Income) using the Small Area Income and Poverty Estimates, and the percent experiencing unemployment (Unemployed) using data from the Bureau of Labor Statistics. Finally, researchers studying health and health disparities have noted the importance of community demographics, including the percent of people aged 65 years or older (65 plus), the percent of the county residents living in a rural setting (Rural), the gender composition (reflected here as the percent of women; Female), the racial characteristics (reflected here as the percent White), and the overall population size (Population). All of these data came from Census Bureau estimates.

### 2.2. Empirical Analysis

The empirical analysis consists of two main steps. First, descriptive statistics were obtained for all variables. Second, we tested the hypotheses through three moderated regression analyses, following Cohen, Cohen, West, and Aiken’s [57] guidelines Models 1a–c include only the control variables. Since the measures for population size and income were skewed, their natural logarithm was used to move these variables closer to the normal distribution which is required for regression analysis. Such a log-transformation is a common procedure [17]. We then standardized the first order effects to reduce multicollinearity [57] and entered them in a second step (Models 2a–c; see also [58]). In the final step (Models 3a–c), we entered the AirPollution × AccesstoPA interaction term and the WaterPollution × AccesstoPA interaction term.

The independent variables of interest and the control variables were tested for multicollinearity prior to estimating the regression models. The correlation matrix (Table 2) indicates that the correlation coefficients between the independent and dependent variables are below the suggested threshold of 0.8 [59], indicating that multicollinearity should not be a concern in the present analyses. All measures of poor health were positively associated with the pollution variables and negatively associated with access to physical activity. The associations between the three measures of poor health and AirPollution were significantly stronger than the associations with WaterPollution, all *t’*s > 9.88 and *p’*s < 0.001.

## 3. Results

### 3.1. Descriptive Statistics

Means, standard deviations, minimums, and maximums are reported in Table 1. In terms of pollution, the counties averaged 9.021 (standard deviation [SD] = 1.987) daily PM2.5 (AirPollution), and 37.1% of the counties had a water violation within the past year. Across counties, the majority of residents had access to physical activity (mean [M] = 62.454, SD = 23.247). Further analysis showed that in 18 of the counties, no residents had access to physical activity, whereas in 49 counties, all residents did. In terms of health, in the average county, almost 18% of the residents reported poor or fair health (M = 17.930, SD = 4.753), they averaged about four days a month of poor physical health (M = 3.991, SD = 0.707), and they averaged over four days of poor mental health (M = 4.168, SD = 0.605).

### 3.2. Hypothesis Testing

Table 3, Table 4 and Table 5 present the results of the regression analyses. We first predicted that AirPollution (H1a) and WaterPollution (H1b) would be positively associated with the residents’ poor health. As seen in Models 2a + b, AirPollution was positively associated with PoorFairHealth (unstandardized coefficient [B] = 0.573, standard error [SE] = 0.042, *p* < 0.001), PhysUnhealthyDays (B = 0.024, SE = 0.007, *p* = 0.001), and MenUnhealthyDays (B = 0.052, SE = 0.007, *p* < 0.001); thus, Hypothesis 1a was supported. Similarly, and in support of Hypothesis 1b, WaterPollution was positively associated with PoorFairHealth (B = 0.162, SE = 0.033, *p* < 0.001), PhysUnhealthyDays (B = 0.033, SE = 0.006, *p* < 0.001), and MenUnhealthyDays (B = 0.043, SE = 0.006, *p* < 0.001).

We also hypothesized that physical activity opportunities would be negatively related to residents’ poor health (H2). As can be seen in Models 2a–c, AccesstoPA has a significant negative association with PoorFairHealth (B = −0.149, SE = 0.044, *p* < 0.01) and PhysUnhealthyDays (B = −0.019, SE = 0.008, *p* < 0.05), but not with MenUnhealthyDays (B = −0.014, SE = 0.008, *p* > 0.05). These results indicate that Hypothesis 2 is only supported for overall poor health and poor physical health, but not for poor mental health.

**Figure 1:**
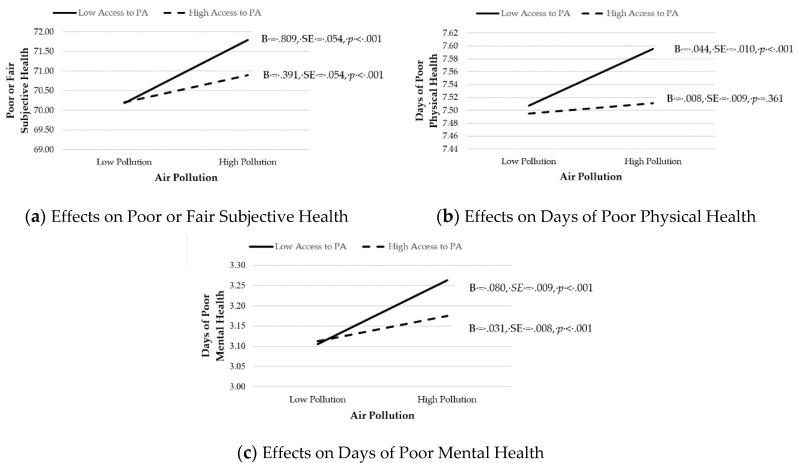
Moderating effects of Access to PA on the relationship between Air Pollution and Poor Health. B = unstandardized coefficient. SE = standard error. PA = physical activity.

We then predicted that AccesstoPA would moderate the effects of AirPollution (H3a) and WaterPollution (H3b) on poor health. Results are shown in Models 3a–c. We found that AccesstoPA moderated the effects of AirPollution on PoorFairHealth (B = −0.209, SE = 0.031, *p* < 0.001), PhysUnhealthyDays (B = −0.018, SE = 0.006, *p* = 0.001), and MenUnhealthyDays (B = −0.024, SE = 0.005, *p* < 0.001). We therefore plotted the interaction and computed simple slopes, per Cohen et al. [56] recommendations, and an illustrative summary is presented in Figure 1. For each health outcome, the effects of AirPollution on poor health outcomes were mitigated when AccesstoPA was high. Thus, Hypothesis 3a was supported. On the other hand, AccesstoPA did not moderate any of the relationships between WaterPollution and poor health (see Models 3a–c). Thus, Hypothesis 3b was not supported.

## 4. Discussion

Existing research has examined the link between pollution, physical activity, and health. While some researchers suggested that outdoor physical activity in polluted conditions can increase the likelihood of health problems [39,41], others point to beneficial outcomes of physical activity on health, which would outweigh the harm caused by pollution [40,42]. However, these mixed results suggest that further investigation is needed to examine the moderating effect of physical activity. In this study, we extended this scholarship to fill this gap to examine the influences of air and water pollution on health outcomes and the moderating role of physical activity opportunities.

To this end, we found that as air and drinking water pollution increased, so too did the poor levels of residents’ health–relationships that have already been well-established in previous research [7,15]. The positive influence of physical activity opportunities on physical health outcomes supports existing research [38], although only few studies have directly examined this link up to now. The non-significant association with the number of mentally unhealthy days evident in this study suggests that access to physical activity only contributes to improvements of less severe mental health indicators like subjective well-being and life satisfaction [17,37]. Further, access to physical activity moderated the relationship between air pollution and residents’ health. However, access to physical activity did not moderate any of the relationships between water pollution and poor overall health, poor physical health, or poor mental health.

In addition, our work makes several contributions to the literature. First, researchers have previously focused on the positive [40] or negative [39] outcomes of physical activity in polluted settings among specific populations. However, there has not been an examination of the influence of pollution on residents’ health and the moderating role of access to physical activity. Our data show a relationship between these factors. Results indicate that access to physical activity moderated the relationship between air pollution and health, but did not moderate the relationship between water pollution and health. We were able to demonstrate that relationship with a national dataset including all U.S. counties, meaning that the study is not based on a sample; it considers the total number of counties. Hence, we provide a richer discussion of the negative influence of air pollution on access to physical activity and the impact on health. These findings add an expanded application by way of a broader population than the previous researchers [39,40,41]. However, our results did not find a moderating effect of access to physical activity on the relationship between water pollution and health outcomes, despite the relationship between water pollution and health outcomes being significant.

Additionally, we also controlled for a variety of factors that have demonstrated to negatively impact health outcomes from previous research. Researchers have previously found that smoker status, obesity, food insecurity, uninsured status, educational level, income, unemployment status, rural setting, gender, racial characteristics, and population size are positively associated with poor health outcomes (e.g., [52,53,54,56]). In our moderated regression analyses, we found the percent of county residents who smoked, the percent of adults classified as obese, the percent of the county residents who were uninsured, the percent of the county residents who had at least some college education, household income, the percent experiencing unemployment, the percent of people aged 65 years or older, the percent of residents living in a rural setting, the gender composition (reflected here as the percent of women), the racial characteristics (reflected here as the percent of Whites), and the overall population size all had a significant relationship with health outcomes, with a few exceptions. We were able to demonstrate the significant and meaningful influence of access to physical activity by controlling for these other factors that can also affect residents’ poor health. Thus, it is important to consider all factors to mitigate poor health outcomes. This includes the necessary infrastructure to support positive health outcomes (i.e., access to physical activity and environmental conditions).

Furthermore, there are several implications based on our findings to inform policymakers and practitioners focused on public and environmental health in and through sport, recreation, and physical activity. The positive outcomes of physical activity on physical health (e.g., [30,31]) and mental health have been well-documented in the literature [13,17]. However, an important aspect that policymakers and practitioners must consider is the state of the surrounding environment, in which, people engage in sport, physical activity, and recreation. Our study suggests that access to physical activity moderates the relationship between air pollution and negative health outcomes. Thus, as policymakers and practitioners address the negative health outcomes of air pollution in their communities they should ensure that their communities provide the corresponding infrastructure facilitating access to physical activity for the resident population. We have demonstrated the positive outcomes that such access can have at lowering the detrimental overall, physical, and mental health outcomes as the result of air pollution.

Notwithstanding, an obvious implication would also be to reduce the levels of air and water pollution across U.S. counties to improve residents’ health. However, the challenge here is that pollution levels have grown over the last decades as a result of industrialization, population growth, urbanization, and unsustainable consumption of natural resources like clean air and water [21,39]. Reducing pollution levels would be associated with significant economic costs for local enterprises and the local economy, resulting in a trade-off decision between environmental improvements and economic growth for local decision-makers. While reducing pollution levels should be a concern for environmental health policy, the role of physical activity opportunities should not be neglected. One significant advantage of the latter is that physical activity patterns of the resident population can be more swiftly changed than structural changes in the local economy. Since physical activity behavior can be immediately implemented [39], it is important that opportunities are available in the residents’ living environment that encourage and facilitate such behavior. Hence, providing physical activity opportunities can represent an effective policy tool for improving residents’ health. In light of the evident effects, combining environmental health and physical activity policies can be a fruitful way for addressing the challenges in public health.

### Limitations and Future Directions

Despite the contributions of our study to the extant literature, theory development, research, and practice, there are potential limitations. First, the data presented determined the access to physical activity and not specific physical activity rates, types of activities (e.g., water-based), or the location of physical activities (e.g., indoor or outdoor activities). A more detailed examination may specifically be useful to more thoroughly examine the moderating effects of the availability of water-based physical activity types on the relationship between water pollution and health outcomes. Examining such types of activity, and more specifically, rates and where these activities take place can better inform policymakers about the influence of pollution on these types and locations of physical activity.

Finally, there are several opportunities for future research. There is a call for researchers to examine and understand the bidirectional relationship between the natural environment with sport, physical activity, and recreation [60,61]. Specifically, in this context, researchers may be interested in examining the detrimental environmental impacts of the causes of climate change on physical activity and general trends in physical activity in response to climate change. That is, how do contributing factors to climate change impacting health outcomes? How are people modifying their behavior in response to climate vulnerabilities (e.g., pollution, increased temperatures, etc.)? What impact do changes in physical activity as a result of climate change have on health outcomes? The dynamic relationship between the natural environment and sport, physical activity, recreation is worthwhile for researchers, practitioners, and policymakers to address the immediate and impending impacts of climate change on these aspects of human activity.

## 5. Conclusions

Overall, the study shows the considerable value of having opportunities to be physically active. In counties where opportunities were plentiful, residents were less likely to report poor or fair health, and they also reported fewer physically unhealthy days. Importantly, physical activity opportunities offset the negative effects of air pollution for all three measures of health. Policy makers are able to draw from this research to improve the health of residents in their communities.

## Figures and Tables

**Table 1:** Variable summary and descriptive statistics.

Variable	Description	*n*	M	SD	Min	Max
AirPollution	Average daily PM2.5	3070	9021	1978	3000	19.700
WaterPollution	Presence of drinking water violations in the county (0 = no; 1 = yes)	3099	0.371	0.483	0.000	1000
AccesstoPA	Percent of county residents with access to physical activity opportunities	3098	62.454	23.247	0.000	100.000
PoorFairHealth	Percent of county residents with poor or fair subjective health ratings.	3104	17.930	4753	8121	40.991
PhysUnhealthyDays	Average number of physically unhealthy days among county residents	3104	3991	0.707	2449	7062
MenUnhealthyDays	Average number of mentally unhealthy days among county residents	3104	4168	0.605	2533	6.14
Smokers	Percent of smokers	3104	17.462	3619	5909	41.491
Obesity	Percent of adults with obesity	3104	32.874	5442	12.400	57.700
FoodInsecure	Percent of food insecure	3104	13.237	3941	2900	36.300
Uninsured	Percent Uninsured	3103	13.592	6250	2683	42.397
SomeCollege	Percent of population with some college	3104	57.804	11.810	15.175	100.000
Population	Number of residents (in thousand)	3104	104.574	335.340	0.088	10,105.518
65plus	Percent of population aged 65 or older	3104	19.311	4701	4830	57.857
Rural	Percent of population rural	3097	59.273	31.044	0.000	100.000
Female	Percent of population that is female	3104	49.859	2273	26.835	56.871
White	Percent of population that is white	3104	76.195	20120	2690	97.890
Income	Median household income (in thousand dollar)	3103	52.754	13.730	25.385	140.382

Notes. M = mean. SD = standard deviation. PM2.5 = fine particulate matter of 2.5 micrometers and smaller.

**Table 2:** Correlation matrix.

Variable	1	2	3	4	5	6	7	8	9
1. Smokers	---								
2. Uninsured	0.093 **	---							
3. SomeCollege	−0.537 **	−0.441 **	---						
4. Unemployed	0.439 **	0.026	−0.382 **	---					
5. 65 plus	−0.137 **	−0.023	−0.057 **	0.028	---				
6. Rural	0.201 **	0.185 **	−0.321 **	0.061 **	0.480 **	---			
7. Female	0.006	−0.103 **	0.246 **	−0.006	0.084 **	−0.177 **	---		
8. White	−0.108 **	−0.472 **	0.264 **	−0.295 **	0.389 **	0.304 **	0.039 *	---	
9. Obesity	0.496 **	0.059 **	−0.372 **	0.239 **	−0.102 **	0.174 **	0.040 *	−0.089 **	---
10. FoodInsecure	0.596 **	0.295 **	−0.424 **	0.535 **	−0.063 **	0.071 **	0.087 **	−0.424 **	0.368 **
11. log_Population	−0.107 **	−0.267 **	0.258 **	−0.008	−0.445 **	−0.778 **	0.277 **	−0.199 **	−0.123 **
12. log_Income	−0.635 **	−0.339 **	0.622 **	−0.436 **	−0.260 **	−0.399 **	0.03	0.169 **	−0.422 **
13. AirPollution	0.314 **	−0.143 **	−0.161 **	0.177 **	−0.289 **	−0.221 **	0.231 **	−0.053 **	0.262 **
14. WaterPollution	−0.055**	0.019	−0.015	0.102 **	−0.083 **	−0.178 **	0.010	−0.119 **	−0.037 *
15. AccesstoPA	−0.300 **	−0.278 **	0.387 **	−0.082 **	−0.179 **	−0.594 **	0.105 **	−0.007	−0.289 **
16. PoorFairHealth	0.728 **	0.414 **	−0.664 **	0.495 **	−0.159 **	0.088 **	0.022	−0.537 **	0.417 **
17. PhysUnhealthyDays	0.811 **	0.216 **	−0.624 **	0.548 **	−0.064 **	0.121 **	0.072 **	−0.273 **	0.391 **
18. MenUnhealthyDays	0.756 **	0.101 **	−0.557 **	0.490 **	−0.018	0.075 **	0.167 **	−0.144 **	0.356 **
	**10**	**11**	**12**	**13**	**14**	**15**	**16**	**17**	**18**
10. FoodInsecure	---								
11. log_Population	−0.048 **	---							
12. log_Income	−0.684 **	0.405 **	---						
13. AirPollution	0.240 **	0.430 **	−0.036 *	---					
14. WaterPollution	0.039 *	0.216 **	0.037 *	−0.004	---				
15. AccesstoPA	−0.261 **	0.522 **	0.447 **	0.000	0.119 **	---			
16. PoorFairHealth	0.708 **	−0.084 **	−0.723 **	0.284 **	0.043 *	−0.341 **	---		
17. PhysUnhealthyDays	0.702 **	−0.033	−0.712 **	0.315 **	0.058 **	−0.290 **	0.891 **	---	
18. MenUnhealthyDays	0.638 **	0.070 **	−0.621 **	0.400 **	0.082 **	−0.234 **	0.781 **	0.934 **	---

Notes. * *p* < 0.05. ** *p* < 0.01.

**Table 3:** Regression results for subjective ratings of Poor or Fair Health.

	Model 1a		Model 2a		Model 3a	
Variable	B	SE	B	SE	B	SE
Smokers	0.551 ***	0.015	0.541 ***	0.015	0.545 ***	0.015
Uninsured	0.068 ***	0.009	0.077 ***	0.009	0.081 ***	0.009
SomeCollege	−0.067 ***	0.004	−0.051 ***	0.004	−0.045 ***	0.004
Unemployed	0.284 ***	0.031	0.287 ***	0.03	0.294 ***	0.03
65plus	−0.069 ***	0.010	−0.045 ***	0.010	−0.041 ***	0.010
Rural	−0.007 ***	0.002	−0.012 ***	0.002	−0.012 ***	0.002
Female	0.173 ***	0.017	0.133 ***	0.016	0.126 ***	0.016
White	−0.080 ***	0.002	−0.085 ***	0.002	−0.086 ***	0.002
Obesity	−0.022 **	0.007	−0.037 ***	0.007	−0.036 ***	0.007
FoodInsecure	0.024	0.014	−0.011	0.014	−0.023	0.014
log_Population	−0.36 ***	0.09	−0.808 ***	0.095	−0.812 ***	0.095
log_Income	−11.041 ***	0.648	−11.58 ***	0.627	−11.679 ***	0.623
AirPollution (AP)			0.573 ***	0.042	0.6 ***	0.042
WaterPollution (WP)			0.162 ***	0.033	0.163 ***	0.032
AccesstoPA (APA)			−0.149 **	0.044	−0.198 ***	0.045
AP × APA					−0.209 ***	0.031
WP × APA					−0.023	0.033

Notes. ** *p* < 0.01. *** *p* < 0.001. B = unstandardized coefficient. SE = standard error.

**Table 4:** Regression results for the average number of physically unhealthy days.

	Model 1b		Model 2b		Model 3b	
Variable	B	SE	B	SE	B	SE
Smokers	0.100 ***	0.003	0.100 ***	0.003	0.101 ***	0.003
Uninsured	0.002	0.002	0.002	0.002	0.003	0.002
SomeCollege	−0.012 ***	0.001	−0.011 ***	0.001	−0.010 ***	0.001
Unemployed	0.069 ***	0.005	0.068 ***	0.005	0.068 ***	0.005
65plus	−0.003	0.002	−0.002	0.002	−0.001	0.002
Rural	0.000	0.000	−0.001 *	0.000	−0.001 **	0.000
Female	0.026 ***	0.003	0.025 ***	0.003	0.024 ***	0.003
White	−0.001	0.000	−0.001 *	0.000	−0.001 *	0.000
ObeseAdults	-0.012 ***	0.001	−0.013 ***	0.001	−0.013 ***	0.001
FoodInsecure	0.023 ***	0.002	0.022 ***	0.002	0.021 ***	0.002
log_Population	0.100 ***	0.015	0.074 ***	0.017	0.075 ***	0.017
log_Income	−1.313 ***	0.111	−1.314 ***	0.110	−1.323 ***	0.110
AirPollution (AP)			0.024 ***	0.007	0.026 ***	0.007
WaterPollution (WP)			0.033 ***	0.006	0.034 ***	0.006
AccesstoPA (PA)			−0.019 *	0.008	−0.024 **	0.008
AP × PA					−0.018 **	0.006
WP × PA					−0.005	0.006

Notes. * *p* < 0.05. ** *p* < 0.01. *** *p* < 0.001.

**Table 5:** Regression results for the average number of mentally unhealthy days.

	Model 1c		Model 2c		Model 3c	
Variable	B	SE	B	SE	B	SE
Smokers	0.082 ***	0.003	0.082 ***	0.003	0.083 ***	0.003
Uninsured	−0.001	0.002	0.000	0.002	0.000 ***	0.002
SomeCollege	−0.014 ***	0.001	−0.012 ***	0.001	−0.011 ***	0.001
Unemployed	0.056 ***	0.005	0.054 ***	0.005	0.054 ***	0.005
65plus	0.004 **	0.002	0.007 ***	0.002	0.007 ***	0.002
Rural	0.000	0.000	−0.001	0.000	−0.001 *	0.000
Female	0.037 ***	0.003	0.034 ***	0.003	0.033 ***	0.003
White	0.003 ***	0.000	0.003 ***	0.000	0.003 ***	0.000
ObeseAdults	−0.010 ***	0.001	−0.012 ***	0.001	−0.012 ***	0.001
FoodInsecure	0.030 ***	0.002	0.027 ***	0.002	0.026 ***	0.002
log_Population	0.189 ***	0.015	0.138 ***	0.016	0.138 ***	0.016
log_Income	−0.503 ***	0.109	−0.527 ***	0.107	−0.539 ***	0.107
AirPollution (AP)			0.052 ***	0.007	0.055 ***	0.007
WaterPollution (WP)			0.043 ***	0.006	0.043 ***	0.006
AccesstoPA (PA)			−0.014	0.008	−0.020 **	0.008
AP × PA					−0.024 ***	0.005
WP × PA					−0.007	0.006

Notes. * *p* < 0.05. ** *p* < 0.01. *** *p* < 0.001.
